# *In vivo* enzymatic digestion of HRV 3C protease cleavage sites-containing proteins produced in a silkworm-baculovirus expression system

**DOI:** 10.1042/BSR20220739

**Published:** 2022-06-14

**Authors:** Jian Xu, Takafumi Nakanishi, Tatsuya Kato, Enoch Y. Park

**Affiliations:** 1 Laboratory of Biotechnology, Green Chemistry Research Division, Research Institute of Green Science and Technology, Shizuoka University, 836 Ohya, Shizuoka 422-8529, Japan; 2 Department of Agriculture, Graduate School of Integrated Science and Technology, Shizuoka University, 836 Ohya, Suruga-ku, Shizuoka 422-8529, Japan

**Keywords:** 3C proteinase, baculovirus expression system, co-infection, silkworm, Tag-free

## Abstract

Baculovirus expression vector system (BEVS) has been recognized as a potent protein expression system in engineering valuable enzymes and vaccines. Various fusion tags facilitate protein purification, leaving the potential risk to influence the target protein's biological activity negatively. It is of great interest to consider removing the additional tags using site-specific proteases, such as human rhinoviruses (HRV) 3C protease. The current study validated the cleavage activity of 3C protease in *Escherichia coli* and silkworm-BEVS systems by mixing the cell or fat body lysates of 3C protein and 3C site containing target protein *in vitro*. Further verification has been performed in the fat body lysate from co-expression of both constructs, showing remarkable cleavage efficiency *in vivo* silkworm larvae. We also achieved the glutathione-S-transferase (GST) tag-cleaved product of the VP15 protein from the White spot syndrome virus after purification, suggesting that we successfully established a coinfection-based recognition-and-reaction BEVS platform for the tag-free protein engineering.

## Introduction

Protein expression and purification have become an essential technology to uncover the protein-involved mechanisms and to produce pharmaceutically relevant products, such as vaccines and drugs [1–3]. Indeed, remarkable progress has been made to improve the various hosts-based protein expression systems either at the small-scale (e.g., laboratory base) or industrial level to meet the demand of the clinical market [2–10]. To shorten the duration from plasmid DNA construction to the final purified protein product, a series of affinity tags (e.g., poly-Histidine, Streptavidin) have been introduced and have become a common strategy in this field [11]. Some affinity tags of higher molecular weight, such as maltose-binding protein (MBP), small ubiquitin-related modifier (SUMO), and glutathione-S-transferase (GST), have been commonly used to enhance the stability or solubility of proteins of interest (POIs) [5,11–16]. These tags generally accelerate the purification efficiency by using affinity chromatography, leaving the potential risk of negatively influencing the biological activity of POIs. Tag-free POIs from various protein expression systems show advantages in protein engineering and animal immunology to avoid tag-specific antibody production. It is of great interest to consider removing the additional tags using site-specific proteases, such as Tobacco Etch Virus (TEV) protease or Human rhinoviruses (HRV) 3C protease (also known as PreScission protease), which are commercially available for research. However, the efficiency of *in vitro* protease-based reactions is condition-dependent (buffer, temperature, etc.) [17–19]. Moreover, the second purification to remove free tags and proteases with further buffer exchange procedures is very time-consuming and possibly results in a significant loss of POIs, either in the amount or overall activity. It is then beneficial to develop an *in vivo* platform to express and digest the POIs, especially when the affinity fusion tag is relatively bigger, i.e., GST [20,21].

Baculovirus vector expression system (BEVS) using lepidopteran insect hosts, either larvae or culture cells, was established decades ago [22–24]. Among others, the BEVS system’s merits are its robustness and flexibility in co-expressing multiply POIs via a co-infection approach with the recombinant baculovirus mixture, through which the protein complex can be expressed and assembled readily *in vivo* [4,25–27]. Several flexible platforms have been established and verified to produce protein complexes *in vivo* and *in vitro* [28–32]. To the best of our knowledge, nine BEVS-derived products, either protein subunits or virus-like particles (VLPs) have been approved for commercial application against human (Cervarix®, Provenge®, Glybera®, and Flublok®) or veterinary (Porcilis® Pesti, BAYOVAC CSF E2®, Circumvent® PCV, Ingelvac CircoFLEX®, and Porcilis® PCV) pathogens [33]. Although the expression and activity were performed in the *Escherichia coli* system for TEV and 3C proteinases [18,19], there are no examples in BEVS and other protein expression systems to generate tag-free or tag-less POIs by co-expressing tagged POIs (i.e., GST-3C-POI) with a digestion enzyme (i.e., 3C protease). Previously, VP15 from white spot syndrome virus (WSSV) has been identified as an antigenic protein against WSSV infection in Kuruma shrimp *Marsupenaeus japonicus*, which was produced in either *E. coli* or silkworm expression system [34]. Compared with the high expression in *E. coli*, the low yield of VP15 in silkworm larvae constrains its potential application [34]. We confirmed in this study that the expression level of the soluble fraction of VP15 could be improved with an additional GST tag at its N-terminus.

Based on these results, we designed and developed a platform where recognition and digestion between GST-3C_site_-VP15-Flag and GST-3C_protease_ coincide in lepidopteran silkworm (*Bombyx mori*) larvae-based BEVS (silkworm-BEVS) *in vivo*. The 3C protease-dependent cleavage activity is confirmed through either *in vitro* mixture in *E. coli* or *in vivo* co-infection in silkworm-BEVS. The VP15-Flag digested from GST-3C-VP15-Flag was also successfully purified, indicating the utilization and application of the current strategy in the production of POIs with low expression levels.

## Materials and methods

### Silkworm larvae and cells

The fourth instar silkworm larvae were purchased from Ehime Sansyu (Ehime, Japan) and carefully reared under a controlled environment (25°C, 65 ± 5% relative humidity) with an artificial diet (Silkmate S2, Nosan, Japan) for ∼5 days to reach fifth instar larvae prepared for viral infections. The cultured silkworm Bm5 cells were passaged in Sf-900II medium (Thermo Fisher Scientific K. K., Tokyo, Japan) supplemented with 10% fetal bovine serum (Gibco, Tokyo, Japan) and 1% antibiotic-antimycotic (Thermo Fisher Scientific K. K., Tokyo, Japan) at 27°C.

### Plasmid and baculovirus constructions of human rhinoviruses (HRV) 3C protease

Based on the genome sequence of HRV (accession number: NC_001490.1), we ordered a synthetic 3C gene from Genewiz (Suzhou, China). The DNA fragment was then inserted into the pET41a (+) vector (Merck, Darmstadt, Germany) to express GST-fused 3C protein (GST-HRV3C) in *E. coli*. The primer set was 3Cpt-FW-BamHI (5′-atcgggatccatgggaccaaacacagaatt-3′) and 3Cpt-RV-XhoI-NoStop (5′-ccggctcgagttgtttctctacaaaatattg-3′) with BamHI and XholI restriction sites, respectively. To avoid a conflict during protein purification for the FLAG-tagged POIs (GST-VP15-FLAG) from silkworm-BEVS, the enterokinase site (DDDDK) was removed from pET41a-GST-HRV3C using inverse PCR [KOD-Plus-Neo DNA polymerase, (Toyobo, Osaka, Japan); 3Cpt-FW 5′-atgggaccaaacacagaatt-3′ and GGGSG-RV 5′-accggagccaccaccggtaccca-3′]. Subsequently, GST-HRV3C^∆FLAG^ was amplified using another primer set (GST-FW 5′-atgtcccctatactaggttattgg-3′ and pET-RV 5′-ggttatgctagttattgctc-3′, XholI digested) and inserted into the StuI/XholI double digested pFastbac-1 vector (Thermo Fisher Scientific, K. K.), termed pFastBac-GST-HRV3C, for generating recombinant *B. mori* nucleopolyhedrovirus (BmNPV) bacmid using BmDH10Bac *E. coil* [32]. All of the sequences were confirmed through DNA sequencing. The resulting bacmid DNA was used for transfecting cultured silkworm Bm5 cells using DMRIE-C transfection reagents (1 μl/2 μg bacmid DNA, Thermo Fisher Scientific K. K.) as described previously [29]. The target protein expression was confirmed in Bm5 cell lysates, and the recombinant viruses used for infecting silkworm larvae were achieved by the series infection method.

### Expression and purification of GST-HRV3C in *E. coli*

The verified plasmids, either GST-VP15-FLAG in pGEX-6P-1 [34] or pET-41a-GST-HRV3C, were transformed into *E. coli* Rosetta-gami B (Merck) by electroporation transformation. Transformed *E. coli* cells were then employed to express corresponding proteins in LB broth induced by adding 0.5 mM isopropyl β-D-1-thiogalactopyranoside (IPTG) at 16°C for 16 h. The cell pellets (6,000 × ***g***, 15 min, 4°C) were washed twice and resuspended in phosphate buffer saline (PBS, pH 7.3) containing 1 × proteinase inhibitor (cOmplete, Mini, EDTA-free Protease Inhibitor Cocktail, Sigma-Aldrich Japan, Tokyo) and 10 μg/ml lysozyme. The whole-cell lysates from disrupted cells by sonication (Vibra-Cell^™^ Ultrasonic Liquid Processors, Sonics & Materials Inc, Newtown, CT, U.S.A.) were centrifuged (10,000 × ***g***, 20 min, 4°C) and the samples of supernatants and precipitates were collected, which were employed for investigating the expressed recombinant proteins. The resulting sodium dodecyl sulfate-polyacrylamide gels (SDS-PAGE) were stained by Coomassie brilliant blue (CBB). Western blotting using anti-His (1:5,000, MBL, Nagoya, Japan) or anti-FLAG (DYKDDDDK) antibodies (1:5,000, MBL, Nagoya, Japan) was performed to identify the expressed proteins before subsequential protein purifications.

As described in our previous report for protein purifications, the clear supernatant containing GST-fused proteins was loaded onto a GST affinity chromatographic column (Glutathione Sepharose 4 Fast Flow, Cytiva, Tokyo, Japan) following the manufacturer’s instructions [34]. In brief, the glutathione resin was pre-equilibrated with 10 × bed volumes of PBS (pH 7.3) before applying cell lysates. Then, 10 × bed volumes of PBS were used for column wash, and elution was done with the elution buffer containing 10 mM glutathione in 50 mM Tris-HCl (pH 8.0). Purified proteins were concentrated and dialyzed against PBS using Amicon Ultra-15 30 K (Merck Japan, Tokyo, Japan). The protein concentration was estimated via BCA assay (Pierce BCA Protein Assay Kit, Thermo Fisher Scientific, K. K.).

### Expression and purification of POIs from silkworm fat body

According to our previous reports, the expression and purification of target proteins in silkworms were performed [29,34]. Briefly, the fat body from silkworm larvae at 5 days post-infection was collected and resuspended in a lysis buffer (100 mM Tris-HCl pH 8.4, 0.15 M NaCl, 1 mM EDTA, 0.1% NP-40, 1 × proteinase inhibitor). The suspensions were sonicated and centrifuged, and the clear supernatants were obtained for analysis by SDS-PAGE followed by CBB staining and Western blotting using anti-His (1:5,000, MBL Nagoya, Japan) or anti-FLAG antibodies (1:5,000, MBL).

The FLAG-tagged proteins were subjected to anti-DDDDK-conjugated protein purification gel (MBL). In brief, the column was pre-equilibrated with 10 × bed volumes of washing buffer containing 300 mM NaCl in 50 mM Tris-HCl (pH 7.5) before applying the fat body lysates, then eluted the proteins by an elution buffer comprised of 0.17 M glycine-HCl buffer (pH 2.3). The eluate was immediately neutralized with PBS (pH 7.3) and concentrated using Amicon Ultra-15 30 K (Merck).

### 3C mediated protein cleavage assay *in vitro* and *in vivo*

To verify the activity of expressed recombinant 3C, the cell lysates from GST-3C-expressing *E. coli* and silkworm fat body were obtained in a commercial 3C cleavage buffer (50 mM Tris-HCl pH 8.0, 150 mM NaCl) supplemented with 0.1% NP-40, since NP-40 does not influence its protease activity (Cat. # 7360, Takara-Bio, Tokyo, Japan). The *E. coli* cell lysate from GST-VP15 and GST-3C was mixed at 1:1 (+) or 1:2 (++) (v/v) and incubated on ice for 4 h, which was subjected to SDS-PAGE or Western blot analysis. Same protocols were applied to silkworm fat body lysate mixture under 1:1 (+), 1:2 (++), and 1:3(+++) (v/v) ratio. Co-expression of 3C protease and VP15 was performed by infecting fifth instar silkworm larvae (day 3) with a 1:1 mixture of each baculovirus at ∼1 × 10^5^ plaque-forming unit (pfu) per larvae. At 5 days post-infection, the silkworm larvae were dissected, and the fat body was collected into the 3C reaction buffer (100 mM Tris-HCl pH 8.4, 0.15 M NaCl, 1 mM EDTA, 0.1% NP-40, 1 × proteinase inhibitor). The clear supernatants from sonicated samples were subjected to SDS-PAGE or Western blotting.

## Results and discussion

### Cleavage activity confirmed from 3C protease expressed in *E. coli*

**Figure 1:**
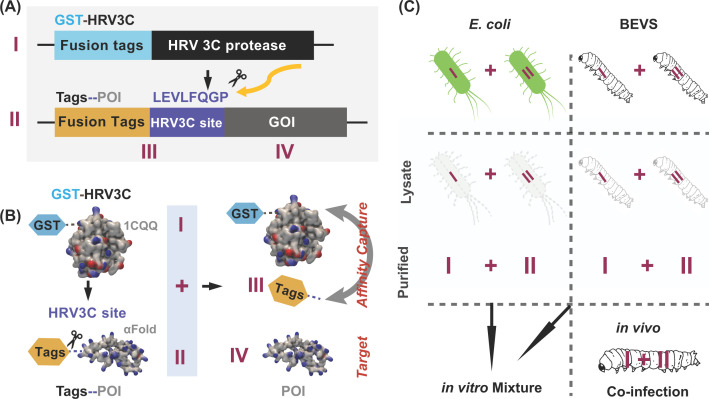
Schematic design of expression and *in vitro* and *in vivo* digestion activity of HRV 3C proteinase in *E. coli* and silkworm-baculovirus expression systems (silkworm-BEVS) (**A**) The expression cassette of HRV 3C proteinase (I) and HRV3C recognition site (LEVLFQGP)-containing the gene of interest (GOI) (II). (**B**) Expressed GST-HRV3C proteinase (I) recognizes the specific 3C-site and future removes the fusion polytags from Tags-GOI (II), resulting in separated Tags (III) and POI (IV) proteins. This reaction could be taken into practice *in vitro* by mixing the purified proteins of I and II, cell lysates from *E. coli* or silkworm-BEVS, or co-expressing I/II together in silkworm via co-infection technique *in vivo* (**C**).

HRV 3C protease recognizes LEVLFQGP and has a precise cleavage between Q and GP residues [36]. It is frequently employed in various protein expression vectors in *E coli*, such as pGEX-6P-1 which was used in this study, to remove GST-tag after protein purifications on-demand. As demonstrated in Figure 1A, we designed two expression cassettes containing the 3C proteinase gene in pET41a (I) and 3C recognition site, including GOI in pGEX-6P-1 (II). The resulting products upon digestion would be convenient for removing the affinity tags (III), leaving only POIs (IV) for subsequential tests (Figure 1B). The digestion condition could occur *in vitro* by mixing the rough or purified cell products from *E. coli* or silkworm fat body tissues infected by recombinant baculoviruses (Silkworm-BEVS). Since BEVS allows co-expression of multiple POIs simultaneously via a co-infection approach, it is then applicable for achieving POIs or protein complexes free of reductant affinity tags before purifications (Figure 1C).

**Figure 2:**
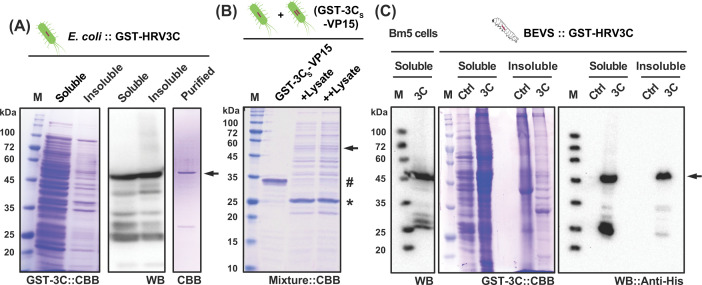
GST-tagged HRV3C protein is soluble and functional in the *E. coli* expression system A GST-tagged HRV3C protein was expressed from pET-41a-HRV3C in Rosetta-gami 2 *E. coli* strain. The IPTG-induced soluble and insoluble cell lysates were loaded and verified in CBB and Western blot using an anti-His antibody. The recombinant 3C enzyme was also purified using GST-column (**A**). The purified GST-VP15 protein (#) containing a 3C-recognition site (3Cs) was mixed with 3C enzyme expressing cell lysate (arrowhead) from A in 1:1 (+) and 1:2 (++), showing fully digested bands for GST-VP15 as indicated by asterisk (*). M: molecular mass markers (**B**). Recombinant GST-HRV3C was expressed in cultured Bm5 cells or silkworm-BEVS. The fat body lysates from virus-infected silkworm were tested against anti-His6 antibody and showed a specific GST-tagged HRV3C band in both soluble (Lysate) and insoluble (Precipitation) forms as indicated with an arrow (**C**).

As the first step of this study, we constructed pET-41a-HRV3C from a synthetic HRV3C DNA fragment and expressed GST-HRV3C in *E. coli*. It shows in Figure 2A that the product was expressed in the correct size (∼53.8 kDa) and could be purified by GST affinity chromatography (∼0.45 mg/L). We adopted one POI, VP15 of WSSV, from our previous study as proof of concept. This protein was potentially interesting regarding its possible use as one vaccine candidate against WSSV infection in Kuruma shrimp *M. japonicus*. In this regard, the quality and quantity of VP15 products are both required for fundamental and applied studies. Upon mixing and incubating the cell lysates of *E. coli* expressing GST-HRV3C or GST-3Csite-VP15 on ice for 4 h, GST-3Csite-VP15 was digested efficiently, and the resulting products as predicted can be clearly seen and verified from SDS-PAGE analysis (Figure 2B), suggesting the GST-3C was active in *E. coli* lysate as compared to previous studies from active MBP-fused 3C [17,18].

### Silkworm-BEVS produces functional 3C protease *in vitro* and *in vivo*

**Figure 3:**
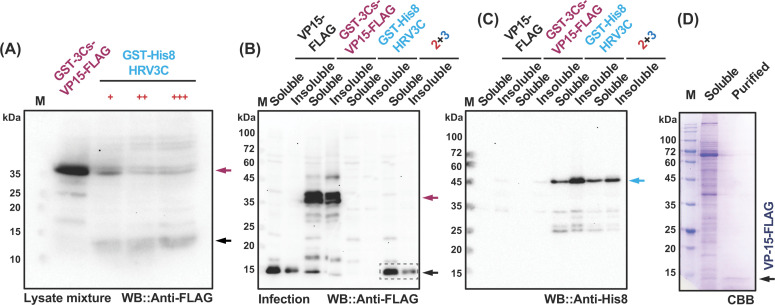
Expression and successful purification of WSSV VP15 protein with low solubility When both fat body lysates were mixed with an increasing amount of GST-3C virus-infected lysates, the GST-VP15 was cleaved efficiently, as demonstrated in (**A**). Dark red and black arrows indicated the original and cleaved bands, respectively. (**B**) The expression verifications of VP15-FLAG protein with or without a GST tag were indicated with black and red arrows, respectively. Upon co-infection of GST-VP15 and GST-3C, the cleaved band as VP15 without a GST-tag showed a similar molecular weight with VP15-FLAG (black arrow). Note that GST-3C is free of FLAG tag, and it thus can be only detected with the anti-His6 antibody (**C**). Successful purification of VP15-FLAG was performed in (**D**). The black arrow indicates the purified VP15 protein. M: molecular mass markers.

Subsequently, we verified the expression of GST-3C (∆FLAG-tag) in either cultured silkworm Bm5 cells (Figure 2C, left panel) or silkworm larvae (Figure 2C, right panel). The correct band was shown around 50 kDa only in the cell or fat body lysate from recombinant 3C baculovirus-infected samples, but not in mock control ones when employing anti-His antibody. To investigate if the expressed 3C protein is active or not *in vitro* and *in vivo*, we then expressed GST-3Csite-VP15-FLAG (GST-VP15) and GST-HRV3C (GST-3C) singly or together in silkworm larvae and collected the fat body tissues which were lysed in 3C reaction buffer with 0.5% NP40 as described in Materials and methods section. We adopted a similar *in vitro* digestion approach when dealing with *E. coli* lysates as an initial step. Upon mixing the fat body lysate of GST-VP15 and GST-3C at 1:1 (+), 1:2 (++), and 1:3 (+++), the original band gradually vanished as the digested product was detected as a faded band at ∼15 kDa after 4 h incubation (Figure 3A). This result indicates that the 3C proteinase was functionally expressed in silkworm-BEVS. Although we did not continue to purify the GST-3C, we can expect a satisfactory amount of 3C protein from silkworm larvae since scaleup expression can be simply performed by increasing the sample size [29,35,37].

It is known that co-infection by virus premixture is one of the merits of the baculovirus expression system [25,28,31,32]. As expected, the additional GST-tag fused to VP15 significantly improved the soluble fraction of VP15 as compared with the relatively low expression level of the VP15-FLAG construct (Figure 3B). When co-expression of GST-VP15 and GST-3C was applied, the shifted band corresponding to the GST-removed product at ∼15 kDa emerged. It is remarkable that the original band at ∼40 kDa from GST-VP15 could merely be detected, implying that the expressed 3C protease (Figure 3B) works well as a protease in silkworm fat body. Compared with the *in vitro* cleavage assay (Figure 3A), the *in vivo* approach seems advantageous as judged by the resulting products shown on Western blot (Figure 3B), possibly a decreased solubility or degradation occurred due to the inappropriate buffer conditions or protein fold issue after *in vitro* cleavage. As demonstrated in Figure 3D, we validated our hypothesis by successfully purifying the cleaved VP15-FLAG protein from *in vivo* co-infection samples. It is known that the traditional approach for tag removal usually occurs after the protein purification, which requires a second purification to remove additional protease. Our established method skips multiple time-consuming processes and directly jumps into the final purification procedure without any loss of target POIs. Further investigations are required to test other POIs to appreciate the potential of our recognition-and-reaction silkworm-BEVS platform. It is also potentially interesting to further balance the expression level between 3C protease and POIs (e.g., ratio control, selective promotors) if the expressed protease is insufficient for a higher amount of POIs.

Previously, we designed an *in vivo* covalent binding platform between SpyTag/SpyCatcher (ST/SC)-tagged protein partners in silkworm-BEVS, termed SpyBEVS [29]. The current result in successful recognition and digestion between 3C proteinase and its recognition residuals is another practical example of protein expression and engineering using the BEVS platform, which can be further applied to other proteinases, such as TEV proteinase [17]. The current platform is also helpful to produce and purify fusion tag-free proteins or protein complexes of interest as needed in antigen and vaccine development. Future study in rational designs (e.g., all-in-one vector or bacmid DNA with various proteinase genes pre-incorporated) [31] and multiple or orthogonal reactions with additional considerations in protein structures combined with the concept from synthetic biology could provide significant progress in silkworm- and BEVS-based biotechnology [28,30,38–40].

## Data Availability

All supporting data related to the studies are provided in the manuscript and available from the corresponding authors upon reasonable request.

## Abbreviations

BEVS: baculovirus expression vector system
CBB: Coomassie brilliant blue
GST: glutathione-S-transferase
HRV: human rhinoviruses
IPTG: isopropyl β-D-1-thiogalactopyranoside
MBP: maltose-binding protein
POI: proteins of interest
SDS-PAGE: sodium dodecyl sulfate-polyacrylamide gels
SUMO: small ubiquitin-related modifier
TEV: tobacco etch virus
